# Chronic High-Fat Diet Exacerbates Sexually Dimorphic *Pomc*^tm1/tm1^ Mouse Obesity

**DOI:** 10.1210/en.2018-00924

**Published:** 2019-03-11

**Authors:** Kristina Hubbard, Avik Shome, Bo Sun, Beau Pontré, Ailsa McGregor, Kathleen G Mountjoy

**Affiliations:** 1 Department of Physiology, Faculty of Medical and Health Sciences, University of Auckland, Auckland, New Zealand; 2 Department of Anatomy and Medical Imaging, Faculty of Medical and Health Sciences, University of Auckland, Auckland, New Zealand; 3 Department of Pharmacy, Faculty of Medical and Health Sciences, University of Auckland, Auckland, New Zealand; 4 Department of Molecular Medicine and Pathology, Faculty of Medical and Health Sciences, University of Auckland, Auckland, New Zealand; 5 Maurice Wilkins Centre for Biodiscovery, University of Auckland, Auckland, New Zealand

## Abstract

Mice with a targeted mutation in the pro-opiomelanocortin (*Pomc*) gene (*Pomc*^tm1/tm1^ mice) are unable to synthesize desacetyl-*α*-MSH and *α*-MSH and they develop obesity when fed chow diet. In this study, we hypothesized that a chronic high-fat (HF) diet exacerbates *Pomc*^tm1/tm1^ mouse obesity. Male and female *Pomc*^wt/wt^ and *Pomc*^tm1/tm1^ mice were fed low-fat (LF) (10 kcal percent fat) or HF (45 kcal percent fat) diets from weaning for 23 weeks. We show that *Pomc*^tm1/tm1^ mouse obesity is sexually dimorphic and exacerbated by an HF diet. Male *Pomc*^tm1/tm1^ mice develop obesity because they are hyperphagic compared with *Pomc*^wt/wt^ mice when fed an LF or HF diet. Female *Pomc*^tm1/tm1^ mice develop obesity when feeding on an LF or HF diet because they exhibit signs of reduced energy expenditure (no change in feed efficiency; body weight gained exceeding energy intake) compared with *Pomc*^wt/wt^ mice. A chronic HF diet exacerbates male *Pomc*^tm1/tm1^ and *Pomc*^wt/wt^ mouse obesity, and the increased energy intake fully accounts for increased weight gain. In contrast, female *Pomc*^wt/wt^ mice are protected from chronic HF diet–induced obesity because they reduce the amount of HF diet eaten, and they appear to increase their energy expenditure (no change in feed efficiency but energy intake exceeding body weight gained). A chronic HF diet exacerbates female *Pomc*^tm1/tm1^ mouse obesity due to impaired ability to reduce the amount of HF diet eaten and apparent impaired HF diet–induced adaptive thermogenesis. Our data show that desacetyl-*α*-MSH and *α*-MSH are required for sexually dimorphic HF diet–induced C57BL/6J obesity. In conclusion, desacetyl-*α*-MSH and *α*-MSH play salutary roles in sexually dimorphic melanocortin obesity and sexually dimorphic HF diet–induced C57BL/6J obesity.

Interactions between genetic and environmental factors underlie the development of obesity and altered metabolism, and diet is one of the main environmental factors that contributes to this disease (1). Melanocortin obesity (caused by functionally impaired melanocortin signaling) is exacerbated by feeding a high-fat (HF) diet (2–7). Recently, we showed that mice with a targeted mutation in the pro-opiomelanocortin (*Pomc*) gene [*Pomc*^tm1/tm1^ mice; C57BL/6J mice genetically modified so that they are unable to synthesize desacetyl-*α*-MSH and *α*-MSH] are hyperphagic and develop obesity when fed a regular chow diet (8). We also showed that the obesity phenotype is exacerbated by acute (4 day) HF diet feeding due to enhanced hyperphagia and reduced energy expenditure, compared with wild-type (WT) littermate (*Pomc*^wt/wt^) mice. However, chronic HF diet feeding has greater relevance to human obesity where excess caloric intake invariably leads to obesity (1). In this study, we tested our hypotheses that desacetyl-*α*-MSH and *α*-MSH protect mice from chronic HF diet–induced obesity and can reverse chronic HF diet–induced obesity in *Pomc*^tm1/tm1^ mice. Understanding which, and how, specific POMC-derived peptides influence energy metabolism when mice are chronically fed an HF diet might hold answers to how obesity can be prevented or treated.

The interaction of an HF diet with melanocortin obesity is complex and dependent on mouse strain background. Overall, the HF diet effects for melanocortin obesity on a C57BL/6J background are additive, over and above, what occurs in WT C57BL/6J mice (3). An HF diet exacerbates obesity in *Pomc*, melanocortin-3 receptor (*Mc3r*), or melanocortin-4 receptor (*Mc4r*) knockout mice (3, 4, 9, 10). Furthermore, an HF diet exaggerates sexually dimorphic melanocortin obesity and altered glucose metabolism. Male, but not female, *Mc3r* knockout mice gain significantly more weight and are hyperphagic when fed an HF diet compared with a low-fat (LF) diet (10). Male, but not female, *Mc3r*-deficient mice fed an HF diet exhibit increased fasting hyperinsulinemia, hyperglycemia, severe glucose intolerance, and insulin resistance in muscle and adipose tissue (10). An HF diet exacerbates fasting hyperinsulinemia for male and female *Mc4r* knockout mice, but this effect is much greater for female compared with male mice. However, only male *Mc4r* knockout mice fed an HF diet, compared with control mice fed an HF diet or *Mc4r* knockout mice fed an LF diet, exhibit HF diet–induced hyperglycemia (10).

HF diet–induced obesity and comorbidity phenotypes in WT rats and mice can also be sexually dimorphic. HF diet–induced rodent obesity is influenced by strain, sex, age, dietary fat content, length of time on diet, and environmental temperature (11). C57BL/6J mice are a commonly used mouse model of HF diet–induced obesity (12, 13) because they rapidly develop obesity with insulin resistance in liver, muscle, and white adipose tissue on an HF diet (11). However, young female, but not male, C57BL/6J mice are protected from HF diet–induced weight gain and metabolic alterations, including hyperglycemia and hyperinsulinemia (14). This is not specific to the C57BL/6J mouse strain because young female, but not male, rats are also protected from HF diet–induced metabolic alterations (15–17). The age when rodents are exposed to an HF diet influences the phenotype; weight gain for male mice fed an HF diet at weaning until 6 to 12 months, but not for male mice fed an HF diet from 8 to 30 weeks, is significantly different from age-matched control mice fed an LF diet (14).

Development of HF diet–induced obesity is progressive and has been divided into early, middle, and late stages (18). Surwit *et al.* (13) showed that after acute exposure (1 week) to an HF diet, mice do not significantly alter body weight, blood glucose, and insulin levels. However, long-term (24 weeks) on an HF diet induces obesity with hyperphagia and insulin resistance (13). HF diet–induced obesity is associated with activation of low-grade inflammation in the hypothalamus and in peripheral organs (19–21). This inflammatory response, which occurs in male but not female rodents (22–24), develops much more rapidly in the hypothalamus than in peripheral tissues and occurs prior to body weight gain (25). Burchfield *et al.* (26) observed a time-resolved deterioration in the function of a multitude of physiological systems as mice became obese feeding on an HF diet. Adaptation to an HF diet is clearly dynamic from the time that rodents first smell and taste the diet. This adaptation appears to be temperature-dependent because female mice, typically resistant to HF diet–induced obesity, develop obesity when housed at thermoneutrality (27).

In this study, we tested our hypothesis that desacetyl-*α*-MSH and *α*-MSH protect mice from chronic HF diet–induced obesity. Male and female *Pomc*^wt/wt^ and *Pomc*^tm1/tm1^ mice were fed LF (10 kcal percent fat) or moderately HF (45 kcal percent fat) diets from weaning for 23 weeks, while housed at 20°C. To assess the dynamic development of obesity, body weight and food intake were measured throughout this period. To assess sexually dimorphic obesity and associated metabolic alterations, body fat, glucose tolerance, and insulin sensitivity were measured after 16 weeks on diet. Body length, fat and organ weights, blood glucose, leptin, insulin, and adiponectin were measured after 23 weeks on diet. We also tested our hypothesis that either desacetyl-*α*-MSH or *α*-MSH can reverse chronic HF diet–induced obesity in *Pomc*^tm1/tm1^ mice.

## Materials and Methods

### Generation of *Pomc*^wt/wt^ and *Pomc*^tm1/tm1^ mice

The mouse model with a targeted *Pomc* mutation that prevents production of desacetyl-*α*-MSH and *α*-MSH was developed previously (8). For this study, a *Pomc*^wt/tm1^ × *Pomc*^wt/tm1^ breeding colony was maintained and offspring were genotyped as previously described (8).

To compare the effects of LF and HF diets on *Pomc*^wt/wt^ and *Pomc*^tm1/tm1^ phenotypes, 10 *Pomc*^wt/wt^ and 9 *Pomc*^tm1/tm1^ breeding pairs were simultaneously paired to produce litters that were born during a 4-day period. All pups were weaned on the same day, and they were randomly assigned to one of eight groups: *Pomc*^wt/wt^ male LF, *Pomc*^wt/wt^ male HF, *Pomc*^tm1/tm1^ male LF, *Pomc*^tm1/tm1^ male HF, *Pomc*^wt/wt^ female LF, *Pomc*^wt/wt^ female HF, *Pomc*^tm1/tm1^ female LF, and *Pomc*^tm1/tm1^ female HF. Each group had three cages with four mice per cage. All mice in a single cage were derived from different mothers, and there were no more than three mice per group from the same mother.

To assess whether central administration of desacetyl-*α*-MSH or *α*-MSH can reverse male *Pomc*^tm1/tm1^ obesity, further litters from the *Pomc*^tm1/tm1^ breeders generated two independent cohorts of male *Pomc*^tm1/tm1^ mice that were fed either LF or HF diets at weaning for 20 to 21 weeks, before undergoing central melanocortin peptide treatment.

### Ethics and animal husbandry

All experimental procedures involving mice at the Vernon Jensen Animal Facility, University of Auckland, were approved by the Auckland University Animal Ethics Committee and conformed to the Animal Welfare Act 1999. Animals were housed on wood chip bedding and maintained at room temperature (20°C) with a 12-hour dark/12-hour light cycle (lights on at 0700 hours in a pathogen-free barrier facility). The breeding mice were fed regular chow (Teklad Global 18% protein rodent diet 2018; Harlan Laboratories, Madison, WI), and the study mice were fed either an LF diet (LF; Research Diets, New Brunswick, NJ; D12450Bi (10 kcal percent fat, 35% sucrose, 3.85 kcal/g)] or an HF diet [HF; Research Diets; D12451i (45 kcal percent fat, 17% sucrose, 4.73 kcal/g)].

### Growth and development

Groups comprising *Pomc*^wt/wt^ LF, *Pomc*^wt/wt^ HF, *Pomc*^tm1/tm1^ LF, and *Pomc*^tm1/tm1^ HF of each sex were weighed at weaning and then four times per week for the next 35 days to assess the early adaptation response to diet. Following this, they were weighed from 35 days until 16 weeks postweaning (three cages per group) or 21 to 23 weeks postweaning (two cages per group) to assess longer-term adaptation to diet. Food intake was recorded for each cage on the days that body weights were recorded. Food intake was measured manually by weighing the food for each cage and then the intake averaged per cage for each day or week. Prior to weighing the food, partly digested food pellets were retrieved from inside the cage and these were included in the food weight.

### Feed efficiency

Energy intake (kilocalories), body weight gained, and feed efficiency (gram body weight gained per kilocalorie energy consumed × 100) were determined for young mice during rapid growth (2 to 4 weeks postweaning) and for older mature mice (12 to 14 weeks postweaning).

### Glucose tolerance tests and insulin tolerance tests

All mice in one cage from each group underwent glucose tolerance tests (GTTs) at 16 weeks postweaning and insulin tolerance tests (ITTs) at 17 weeks postweaning. Mice were fasted overnight (∼16 hours) and then injected with 1 g/kg body weight glucose (20% dextrose; Health Support, Auckland, New Zealand) IP for a GTT or injected IP with 0.5 U/kg body weight insulin (human recombinant; Roche Diagnostics) for an ITT. Tail vein blood was assayed for glucose at time 0 and then at 30, 60, 90, 120, 150, and 180 minutes after glucose or insulin administration using an Accu-Chek blood glucose meter.

### Body composition

MRI analysis was conducted at ∼20 weeks postweaning on all mice that underwent GTTs and ITTs. MRI was used to assess body composition of *Pomc*^wt/wt^ and *Pomc*^tm1/tm1^ mice. MRI was performed using a 4.7 Tesla horizontal bore magnet interfaced with a UNITY INOVA spectrometer (Agilent Technologies, Santa Clara, CA) using a three-point Dixon acquisition, as previously described (8). These mice were euthanized following MRI without having blood or tissues collected.

### Blood glucose, body length, tail length, and fat pad and organ weights

All mice in the remaining two cages from each group were euthanized between 21 and 23 weeks postweaning. Fasting blood was collected from mice prior to euthanization. Blood glucose was measured on tail vein blood using an Accu-Chek blood glucose meter (Roche Diagnostics). Blood collected using Goldenrod animal lancets (MEDIpoint International, Mineola, NY) was added to ice-cold EDTA Microtainer tubes and centrifuged at 4000*g* for 10 minutes at 4°C. Plasma was then transferred to low-bind Eppendorf tubes and used to measure insulin, leptin, and adiponectin levels.

Mouse body and tail lengths were recorded. Fat pads and organs were dissected and weighed prior to either fixation in formalin or 4% paraformaldehyde, or snap freezing on dry ice.

### Plasma insulin, leptin, and adiponectin measurements

Plasma insulin and leptin were measured using a Milliplex MAP kit with a mouse metabolic magnetic bead panel [catalog no. MMHMAG-44K (28), Millipore Corporation, Bedford, MA]. Plasma insulin was also measured by ELISA [catalog no. EZRMI-13K (29), Millipore Corporation]. Adiponectin was measured using a Milliplex MAP kit mouse adiponectin magnetic bead panel single plex [catalog no. MADPNMAG-70K-0 (30), Millipore Corporation]. Mean interassay and intra-assay coefficient of variation (CV) values for an insulin or leptin Milliplex MAP kit are 7% and 5%, respectively, and the assay standards range from 0.069 ng/mL to 50 ng/mL. Mean interassay and intra-assay CV values for insulin ELISA are 18% and 8%, respectively, and the assay standards range from 0.2 ng/mL to 10 ng/mL. Mean interassay and intra-assay CV values for an adiponectin Milliplex MAP kit are 11% and 3%, respectively, and the assay standards range from 12.2 pg/mL to 12,500 pg/mL.

### Fat cell size

Retroperitoneal, visceral, subcutaneous, and gonadal adipocyte sizes were determined using semiautomated morphometry with two ×20 images of hematoxylin and eosin–stained paraffin sections (10 μm) for each tissue sample. Images were captured on a Zeiss Axioskop 2 microscope. Adipocytes (69 to 475 cells per tissue) were analyzed using ImageJ (31).

### Central melanocortin peptide treatment

Saline vehicle (control), *α*-MSH, or desacetyl-*α*-MSH was administered centrally to male *Pomc*^tm1/tm1^ mice continuously during 14 days using Alzet^®^ osmotic mini pumps (model 1002; Bio-Scientific, Kirrawee, NSW, Australia) and a method previously described (8). The mice aged ∼21 to 22 weeks at the start of treatment were fed LF or HF diets from weaning. *α*-MSH and desacetyl-*α*-MSH were purchased from Bachem (Bubendorf, Switzerland). The pumps delivered 5 μg of either peptide/25 g of mouse body weight/d, a dose that we previously showed consistently and significantly reduces male or female *Pomc*^tm1/tm1^ body weight (8). Mice were group housed (three to six mice per cage), and all mice in each cage received vehicle, desacetyl-*α*-MSH, or *α*-MSH. The experiment was repeated twice with independent cohorts of mice. Individual body weights and food intake for each cage were monitored daily. All mice were euthanized at 14 days of treatment, and nonfasting blood was collected from mice prior to euthanization. Mouse body and tail lengths were recorded. Fat pads and organs were dissected and weighed prior to either fixation in formalin or 4% paraformaldehyde, or snap freezing on dry ice.

### Statistical analysis

GraphPad Prism 6 software (GraphPad Software, San Diego, CA) was used to perform all statistical analyses. Two-way repeated or nonrepeated measures ANOVA with a Tukey, Dunnett, or Sidak *post hoc* analysis was used to compare the effects of LF and HF diets on *Pomc*^wt/wt^ and *Pomc*^tm1/tm1^ phenotypes (body weight and food intake). GTT and ITT significant differences were determined using two-way or one-way (area under the curve) ANOVA and a Tukey *post hoc* test. Significant differences for percent body fat, blood glucose, insulin, leptin, adiponectin, fad pad weight, organ weights, and fat cell size were determined using two-way ANOVA and a Tukey *post hoc* test. Significant differences between groups administered saline, desacetyl-*α*-MSH, or *α*-MSH were determined using two-way repeated measures ANOVA and Dunnett *post hoc* analysis. *P* < 0.05 was considered statistically significant. Data are presented as mean ± SEM.

## Results

### An HF diet exacerbates male and female *Pomc*^tm1/tm1^ mouse growth, but only exacerbates male *Pomc*^wt/wt^ mouse growth

**Figure 1.**
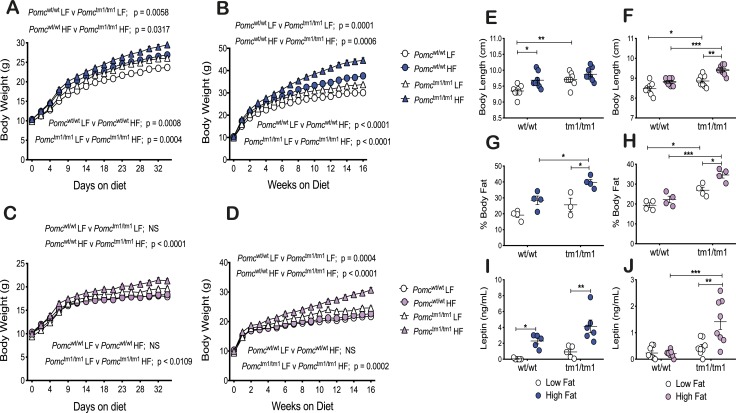
An HF diet exacerbates male and female *Pomc*^tm1/tm1^ mouse increased growth and obesity whereas *Pomc*^wt/wt^ mice exhibit sexually dimorphic HF diet–induced growth and obesity. (A and B) Male body weight. (C and D) Female body weight. (A and C) Body weights were assessed every 3 d for the first 35 d of an LF or HF diet from weaning. Data are shown as mean ± SEM (n = 12). Significant differences for body weights were determined using a two-way repeated measures ANOVA. *F* statistics and Sidak *post hoc* analysis data are shown in an online repository (32) and Table 2. (B and D) Body weights were assessed weekly during 16 wk of an LF or HF diet from weaning. Data are shown as mean ± SEM (n = 12). Significant differences for body weights were determined using a two-way repeated measures ANOVA. *F* statistics and Sidak *post hoc* analysis data are shown in an online repository (32) and Table 2. (E, G, and I) Male phenotype. (F, H, and J) Female phenotype. (E and F) Body length was measured at 21 to 23 wk for mice fed an LF or HF diet from weaning. Data are shown as mean ± SEM (n = 7 to 8). Significant differences were determined using a two-way ANOVA and Tukey *post hoc* test. *P < 0.05; **P < 0.01; ***P < 0.001. (G and H) Percentage body fat was calculated from six MRI Dixon images per mouse. Data are shown as mean ± SEM (n = 3 to 4). Representative images are shown in an online repository (32). Significant differences were determined using a two-way ANOVA and Tukey *post hoc* test. *P < 0.05; ***P < 0.001. (I and J) Fasting plasma leptin measured using a Milliplex MAP kit assay at 21 to 23 wk for mice fed an LF or HF diet from weaning. Data are shown as mean ± SEM (n = 5 to 8). Significant differences were determined using a two-way ANOVA and Tukey *post hoc* test. *P < 0.05; **P < 0.01; ***P < 0.001.

Genotype and diet affect body weight of male mice, with diet influencing body weight at an earlier age than genotype. Body weights for male *Pomc*^wt/wt^ and *Pomc*^tm1/tm1^ mice fed an HF diet were significantly greater beginning at 7 days (*P* < 0.01 and *P* < 0.05, respectively), compared with an LF diet [Fig. 1A; an online repository (32)]. Body weights for male *Pomc*^tm1/tm1^ mice were significantly greater than were body weights for *Pomc*^wt/wt^ mice beginning 16 days (*P* < 0.05) and 25 days (*P* < 0.05) after feeding an LF or HF diet, respectively (Fig. 1A) (32). The increased male body weights associated with genotype or diet continued to increase during 16 weeks (Fig. 1B) (32).

Genotype affected growth of female mice, but in contrast to male mice, the diet effect was dependent on genotype and the genotype effect occurred earlier (∼11 days) (*P* < 0.05) on an HF diet compared with an LF diet (∼5 weeks) (*P* < 0.05) (Fig. 1C and 1D) (32). However, this early genotype effect was likely a result of female *Pomc*^wt/wt^ mice being resistant to HF diet–induced increased body growth, similar to observations reported by others (14). Body weights for female *Pomc*^tm1/tm1^ mice fed an HF diet were significantly greater beginning at 28 days (*P* < 0.05) compared with mice fed an LF diet (Fig. 1C). The increased female body weights associated with genotype or diet continued to gradually increase during 16 weeks (Fig. 1D) (32).

### An HF diet exacerbates female *Pomc*^tm1/tm1^ mouse increased body length and induces a similar trend for male *Pomc*^tm1/tm1^ mice, and an HF diet significantly induces male *Pomc*^wt/wt^ increased body length and induces a similar trend for female *Pomc*^wt/wt^ mice

Our data support previous observations showing that both melanocortin obesity and HF diet–induced obesity in C57BL6/J mice are associated with increased mouse linear growth (8, 20). Similar to our previous observations (8), male *Pomc*^tm1/tm1^ mice were 3.9% longer than male *Pomc*^wt/wt^ mice (*P* < 0.01) when fed an LF diet (Fig. 1E). An HF diet induced a significant 3.6% increase in body length for male *Pomc*^wt/wt^ mice (*P* < 0.05), as well as a trend for increased body length for male *Pomc*^tm1/tm1^ mice (Fig. 1E). Similar to our previous observations (8), female *Pomc*^tm1/tm1^ mice were 4.5% longer than female *Pomc*^wt/wt^ mice (*P* < 0.05) when mice were fed an LF diet (Fig. 1F). An HF diet exacerbated the female *Pomc*^tm1/tm1^ increased body length to 6.8% longer than female *Pomc*^wt/wt^ mice (*P* < 0.001), and there appeared to be a trend for an HF diet to increase female *Pomc*^wt/wt^ body length (Fig. 1F). An HF diet did not affect tail length for male or female *Pomc*^wt/wt^ or *Pomc*^tm1/tm1^ mice.

### An HF diet exacerbates male and female *Pomc*^tm1/tm1^ mouse obesity

Quantitative MRI analysis at ∼17 weeks postweaning on mice fed an HF diet compared with an LF diet revealed that an HF diet induced significant increases in fat mass in male and female *Pomc*^tm1/tm1^ mice (*P* < 0.05) (Fig. 1G and 1H) (32). HF diet–induced increased fat mass was also significant for male *Pomc*^wt/wt^ mice using an unpaired *t* test (*P* < 0.05). Male (*P* < 0.05) and female (*P* < 0.001) *Pomc*^tm1/tm1^ mice had significantly increased body fat compared with *Pomc*^wt/wt^ mice when they were fed an HF diet, and female *Pomc*^tm1/tm1^ mice had significantly increased fat mass compared with female *Pomc*^wt/wt^ mice (*P* < 0.05) when mice were fed an LF diet (Fig. 1G and 1H). There was an apparent trend for male *Pomc*^tm1/tm1^ mice to show increased body fat compared with *Pomc*^wt/wt^ for mice fed an LF diet (Fig. 1G).

The HF diet–induced increased fat masses are reflected in increased fasting plasma leptin. An HF diet significantly increased fasting plasma leptin levels for male *Pomc*^wt/wt^ (*P* < 0.05), male *Pomc*^tm1/tm1^ (*P* < 0.01), and female *Pomc*^tm1/tm1^ (*P* < 0.01) mice compared with respective mice fed an LF diet (Fig. 1I and 1J). Fasting plasma leptin for female *Pomc*^tm1/tm1^ mice compared with *Pomc*^wt/wt^ mice fed an HF diet was significantly increased (*P* < 0.0001), but significance was not reached for male *Pomc*^tm1/tm1^ mice compared with *Pomc*^wt/wt^ mice fed an HF diet.

### Increases in adipose tissue depot masses reflect HF diet exacerbation of male and female *Pomc*^tm1/tm1^ mouse obesity, as well as HF diet–induced male *Pomc*^wt/wt^ mouse obesity

**Figure 2.**
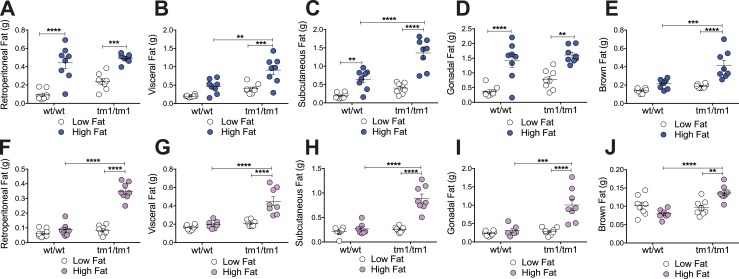
An HF diet compared with an LF diet increases male and female *Pomc*^tm1/tm1^ mouse retroperitoneal, visceral, subcutaneous, gonadal, and brown adipose tissue, specifically increases male *Pomc*^wt/wt^ retroperitoneal, subcutaneous, and gonadal adipose tissue, and has no effect on female *Pomc*^wt/wt^ adipose tissue mass. (A–E) Male and (F–J) female *Pomc*^wt/wt^ and *Pomc*^tm1/tm1^ adipose tissue weights. Retroperitoneal, visceral, subcutaneous, gonadal, and brown fat levels measured at 21 to 23 wk postweaning for mice fed either an LF or HF diet from weaning are shown. Data are shown as mean ± SEM (n = 7 to 8). Significant differences were determined using a two-way ANOVA and Tukey *post hoc* test. **P < 0.01; ***P < 0.001; ****P < 0.0001.

Depot-specific fat storage impacts comorbidities associated with obesity (33). To determine whether the HF diet exacerbation of *Pomc*^tm1/tm1^ mouse obesity and/or *Pomc*^tm1/tm1^ mouse obesity are associated with depot-specific fat storage, specific adipose tissue depots were dissected, weighed, and cell size was quantitated. HF diet exacerbation of male and female *Pomc*^tm1/tm1^ mouse obesity was associated with significantly increased retroperitoneal, visceral, subcutaneous, gonadal, and brown fat weights (Fig. 2A–2J). In contrast, HF diet–induced male *Pomc*^wt/wt^ mouse obesity was associated with significantly increased retroperitoneal, subcutaneous, and gonadal fat weights, but not with visceral and brown fat weights (Fig. 2A–2E). As expected, an HF diet did not significantly affect any fat pad weights for HF diet–resistant female *Pomc*^wt/wt^ mice (Fig. 2F–2J).

In contrast to similar HF diet effects on male and female *Pomc*^tm1/tm1^ fat depot masses, genotype effects on fat pad depot weights appeared to be sexually dimorphic. Male *Pomc*^tm1/tm1^ mice compared with *Pomc*^wt/wt^ mice had significantly increased visceral, subcutaneous, and brown adipose depots when fed an HF diet (Fig. 2B, 2C, and 2E), with no significant differences observed for retroperitoneal or gonadal adipose tissue depot weights (Fig. 2A and 2D). In contrast, female *Pomc*^tm1/tm1^ mice compared with *Pomc*^wt/wt^ mice had significantly increased retroperitoneal, visceral, subcutaneous, gonadal, and brown fat depot weights when fed an HF diet (Fig. 2F–2J). Hence, an HF diet and genotype appeared to differentially increase male *Pomc*^tm1/tm1^ mouse fat depot masses, whereas an HF diet and genotype similarly increased all female *Pomc*^tm1/tm1^ mouse adipose tissue depot weights.

Diet and genotype also differentially affect male adipose tissue cell size. An HF diet compared with an LF diet significantly increased male *Pomc*^wt/wt^ retroperitoneal fat cell size (LF, 1520 ± 212 vs HF, 3775 ± 345; *P* < 0.05) but not subcutaneous fat cell size (LF, 982 ± 117; HF, 893 ± 260). An HF diet did not significantly affect male *Pomc*^tm1/tm1^ retroperitoneal (LF, 2861 ± 142 vs HF, 2718 ± 765) or subcutaneous (LF, 1060 ± 161 vs HF, 2168 ± 424) fat cell size. However, the *Pomc*^tm1/tm1^ genotype significantly increased male subcutaneous fat cell size (*Pomc*^wt/wt^ HF, 893 ± 260 vs *Pomc*^tm1/tm1^ HF, 2168 ± 424, *P* < 0.05) but not male retroperitoneal fat cell size (*Pomc*^wt/wt^ HF, 3775 ± 345 vs *Pomc*^tm1/tm1^ HF, 2718 ± 765) compared with *Pomc*^wt/wt^ mice, when mice were fed an HF diet. Overall, an HF diet appeared to favor increasing retroperitoneal over subcutaneous fat cell size for male *Pomc*^wt/wt^ mice, whereas the male *Pomc*^tm1/tm1^ genotype appeared to favor increasing subcutaneous over retroperitoneal fat cell size. These data support potential differences in fat storage location for diet- and genotype-driven obesity in male mice.

### Chronic HF diet–exacerbated male and female *Pomc*^tm1/tm1^ mouse obesity is not a result of HF diet–exacerbated hyperphagia

**Figure 3.**
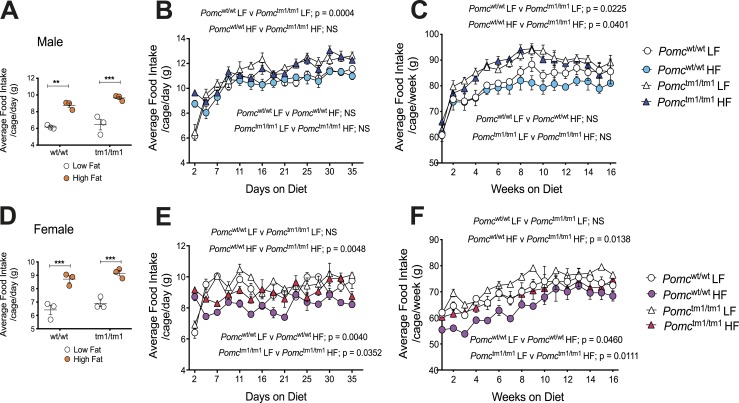
A chronic HF diet compared with an LF diet does not exacerbate hyperphagia in male or female *Pomc*^wt/wt^ or *Pomc*^tm1/tm1^ mice but reduces grams of food eaten by female *Pomc*^wt/wt^ and *Pomc*^tm1/tm1^ mice. (A–C) Male food intake. (D–F) Female food intake. (A and D) Grams of food eaten for the first 2 d after diet introduced at weaning for 12 *Pomc*^wt/wt^ mice (three cages) and 12 *Pomc*^tm1/tm1^ mice (three cages). Data are shown as mean ± SEM (n = 3) and significant differences were determined using a two-way ANOVA and Sidak *post hoc* analysis. **P < 0.01; ***P < 0.001. (B and E) Average food intake per cage per day during 35 d is shown for 12 male (three cages) and 12 female (three cages) mice fed either an LF or HF diet. Data are shown as mean ± SEM (n = 3). Significant differences for food intake were determined using a two-way repeated measures ANOVA. *F* statistics and Sidak *post hoc* analysis data are shown in an online repository (32). (C and F) Average food intake per cage per day during 16 wk is shown for 12 male (three cages) and 12 female (three cages) mice fed either an LF or HF diet. Data are shown as mean ± SEM (n = 3). Significant differences for food intake were determined using a two-way repeated measures ANOVA. *F* statistics and Sidak *post hoc* analysis data are shown in an online repository (32).

We measured food intake to determine whether HF diet–induced hyperphagia contributes to HF diet exacerbation of *Pomc*^tm1/tm1^ mouse obesity (Fig. 3A–3F). An acute HF diet is well known to stimulate an increase in grams of food eaten for rodents (3, 34, 35), and we observed this for male and female *Pomc*^wt/wt^ and *Pomc*^tm1/tm1^ mice previously (8). Male and female *Pomc*^wt/wt^ and *Pomc*^tm1/tm1^ mice ate significantly more grams of food for the first 2 days after an HF diet was introduced compared with an LF diet (Fig. 3A and 3D) (32). However, 4 days after diet was introduced and for the next 16 weeks, the grams of food eaten by male *Pomc*^wt/wt^ mice fed either an LF or HF diet were similar, as were the grams of food eaten by male *Pomc*^tm1/tm1^ mice fed either an LF or HF diet (Fig. 3B and 3C) (32). In contrast, genotype significantly affected male food intake independent of diet. Male *Pomc*^tm1/tm1^ mice ate significantly more grams of food compared with male *Pomc*^wt/wt^ mice when fed either an LF or HF diet (Fig. 3B and 3C) (32).

In contrast with male mice, a chronic HF diet reduced grams of food eaten by both female *Pomc*^wt/wt^ and *Pomc*^tm1/tm1^ mice, compared with an LF diet (Fig. 3E and 3F) (32). This reduction in food eaten was greater for *Pomc*^wt/wt^ compared with *Pomc*^tm1/tm1^ mice, and this explains why female *Pomc*^tm1/tm1^ mice are hyperphagic compared with *Pomc*^wt/wt^ mice. Female *Pomc*^tm1/tm1^ mice ate significantly more grams of food compared with female *Pomc*^wt/wt^ mice, but only when mice were fed an HF diet (Fig. 3E and 3F) (32).

### Feed efficiency is not altered by *Pomc*^tm1/tm1^ obesity or chronic HF diet exacerbation of *Pomc*^tm1/tm1^ mouse obesity

Feed efficiency (body weight gained per kilocalorie consumed) was not significantly different between *Pomc*^wt/wt^ and *Pomc*^tm1/tm1^ mice for males (Table 1) or females (Table 2) during rapid growth (14 to 28 days postweaning) or for mature mice (12 to 14 weeks postweaning). Feed efficiency was also not significantly different between LF and HF diet–fed *Pomc*^wt/wt^ or *Pomc*^tm1/tm1^ male or female mice (Tables 1 and 2).

**Table 1.** Energy Consumed, Body Weight, and Feed Efficiency for Male *Pomc*^wt/wt^ and *Pomc*^tm1/tm1^ Mice

	*Pomc* ^wt/wt^ LF	*Pomc* ^wt/wt^ HF	*Pomc* ^tm1/tm1^ LF	*Pomc* ^tm1/tm1^ HF
Fourteen to 28 d postweaning				
Energy consumed, kcal	61.78 ± 0.26	75.48 ± 2.41^*a*^	69.01 ± 0.36	82.39 ± 1.78^*b*^
Body weight gained, g	3.59 ± 0.05	4.16 ± 0.11	4.47 ± 0.45	5.15 ± 0.28
% Feed efficiency (g body weight gained/kcal consumed × 100)	5.81 ± 0.06	5.52 ± 0.07	6.47 ± 0.63	6.23 ± 0.21
Energy intake on day 28 (kcal/mouse/d)	10.41 ± 0.08	12.52 ± 0.50^*a*^	11.65 ± 0.30	13.62 ± 0.33^*b*^
Body weight at 28 d, g	22.42 ± 0.31	25.58 ± 0.65^*a*^	24.88 ± 0.16^*c*^	27.66 ± 0.22^*b*^
Twelve to 14 wk postweaning				
Energy consumed, kcal	165.77 ± 4.46	193.57 ± 4.54^*a*^	174.20 ± 0.66	210.85 ± 2.48^*b*^
Body weight gained, g	1.32 ± 0.15	1.95 ± 0.38	1.49 ± 0.18	2.50 ± 0.27
% Feed efficiency (g body weight gained/kcal consumed × 100)	0.79 ± 0.08	1.01 ± 0.19	0.85 ± 0.10	1.19 ± 0.13
Energy intake at week 14 (kcal/mouse/wk)	82.07 ± 2.48	96.56 ± 3.26^*a*^	86.97 ± 1.21	104.26 ± 1.46^*b*^
Body weight at 14 wk, g	29.82 ± 0.19	36.63 ± 1.15^*a*^	33.17 ± 0.30	43.62 ± 0.42*^b^*^*,*^*^d^*

Energy consumed, body weight gained, and percentage feed efficiency were calculated for mice fed either an LF or HF diet from weaning. Data were calculated for a period of rapid growth (14 to 28 d postweaning) and at maturity (12 to 14 wk postweaning) and are shown as mean ± SEM for three cages, each housing four mice. Significant differences were determined using a two-way ANOVA and Tukey multiple comparison test.

^a^ *Pomc* ^wt/wt^ LF vs *Pomc*^wt/wt^ HF (*P* < 0.01, energy consumed 14 to 28 d, body weight at 28 d, energy consumed 12 to 14 wk, body weight at 14 wk; *P* < 0.05, energy intake at day 28, energy intake at 14 wk).

^b^ *Pomc* ^tm1/tm1^ LF vs *Pomc*^tm1/tm1^ HF (*P* < 0.01, energy consumed 14 to 28 d, energy consumed 12 to 14 wk, energy intake at 14 wk; *P* < 0.05, body weight at 28 d and energy intake at day 28; *P* < 0.0001, body weight at 14 wk).

^c^ *Pomc* ^wt/wt^ LF vs *Pomc*^tm1/tm1^ LF (*P* < 0.05, body weight at 28 d).

^d^ *Pomc* ^wt/wt^ HF vs *Pomc*^tm1/tm1^ HF (*P* < 0.01, body weight at 14 wk).

**Table 2.** Energy Consumed, Body Weight, and Feed Efficiency for Female *Pomc*^wt/wt^ and *Pomc*^tm1/tm1^ Mice

	*Pomc* ^wt/wt^ LF	*Pomc* ^wt/wt^ HF	*Pomc* ^tm1/tm1^ LF	*Pomc* ^tm1/tm1^ HF
Fourteen to 28 d postweaning				
Energy consumed, kcal	52.6 ± 0.31	57.56 ± 0.66^*a*^	54.20 ± 0.38	64.36 ± 0.67*^b^*^*,*^*^c^*
Body weight gained, g	0.84 ± 0.39	0.86 ± 0.17	1.08 ± 0.10	1.90 ± 0.26
% Feed efficiency (g body weight gained/kcal consumed × 100)	1.60 ± 0.76	1.48 ± 0.27	1.99 ± 0.18	2.94 ± 0.37
Energy intake on day 28 (kcal/mouse/d)	9.62 ± 0.10	9.73 ± 0.13	9.18 ± 0.40	10.74 ± 0.27^*b*^
Body weight at 28 d, g	17.98 ± 0.19	17.75 ± 0.19	18.81 ± 0.24	20.60 ± 0.45*^b^*^*,*^*^c^*
Twelve to 14 wk postweaning				
Energy consumed, kcal	145.13 ± 3.52	167.91 ± 2.80^*a*^	148.13 ± 1.95	172.20 ± 5.09^*b*^
Body weight gained, g	0.87 ± 0.11	1.02 ± 0.06	0.31 ± 0.19	1.81 ± 0.48^*b*^
% Feed efficiency (g body weight gained/kcal consumed × 100)	0.60 ± 0.08	0.61 ± 0.03	0.20 ± 0.12	1.04 ± 0.27
Energy intake at week 14 (kcal/mouse/wk)	72.43 ± 0.79	82.32 ± 2.36^*a*^	74.23 ± 0.57	84.57 ± 2.36^*b*^
Body weight at 14 wk, g	21.26 ± 0.35	22.15 ± 0.19	23.76 ± 0.71	29.17 ± 1.04*^b^*^*,*^*^c^*

Energy consumed, body weight gained, and percentage feed efficiency were calculated for mice fed either an LF or HF diet from weaning. Data were calculated for a period of rapid growth (14 to 28 d postweaning) and at maturity (12 to 14 wk postweaning) and are shown as mean ± SEM for three cages, each housing four mice. Significant differences were determined using a two-way ANOVA and Tukey multiple comparison test.

^a^ *Pomc* ^wt/wt^ LF vs *Pomc*^wt/wt^ HF (*P* < 0.01, energy consumed 14 to 28 d; *P* < 0.05, energy consumed 12 to 14 wk).

^b^ *Pomc* ^tm1/tm1^ LF vs *Pomc*^tm1/tm1^ HF (*P* < 0.0001, energy consumed 14 to 28 d; *P* < 0.05, body weight at 28 d and energy intake at day 28, energy consumed and body weight gained 12 to 14 wk, energy intake at 14 wk; *P* < 0.01, body weight at 14 wk).

^c^ *Pomc* ^wt/wt^ HF vs *Pomc*^tm1/tm1^ HF (*P* < 0.001, energy consumed 14 to 28 d, body weight at 14 wk; *P* < 0.01, body weight at 28 d).

### Increased energy intake fully accounts for increased young, but not older, male and not for young or older female *Pomc*^tm1/tm1^ mouse body weight compared with *Pomc*^wt/wt^ mice

For mice at 28 days postweaning, energy intake and body weight for male *Pomc*^tm1/tm1^ mice significantly increased compared with *Pomc*^wt/wt^ mice by 11.9% (11.65 ± 0.30 vs 10.41 ± 0.08 kcal/mouse/d; *P* < 0.01) and 11.0% (24.88 ± 0.16 vs 22.42 ± 0.31 g; *P* < 0.05), respectively, when mice were fed an LF diet and by 8.8% (13.62 ± 0.33 vs 12.52 ± 0.50 kcal/mouse/d; *P* < 0.01) and 8.1% (27.66 ± 0.22 vs 25.58 ± 0.65 g; *P* < 0.05), respectively, when mice were fed an HF diet (Table 1). However, for older male mice at 14 weeks postweaning, energy intake and body weight for *Pomc*^tm1/tm1^ mice increased compared with *Pomc*^wt/wt^ mice by 6.0% [86.97 ± 1.21 vs 82.07 ± 2.48 kcal/mouse/wk; not significant (NS)] and 11.2% (33.17 ± 0.30 vs 29.82 ± 0.19 g; NS), respectively, when mice were fed an LF diet and by 8.0% (104.26 ± 1.46 vs 96.56 ± 3.26 kcal/mouse/wk; *P* < 0.01) and 19.1% (43.62 ± 0.42 vs 36.63 ± 1.15 g; *P* < 0.01), respectively, when mice were fed an HF diet (Table 1). There were no significant differences for feed efficiency.

For female mice at 28 days postweaning, energy intake and body weight for *Pomc*^tm1/tm1^ mice were not significantly different from *Pomc*^wt/wt^ mice when mice were fed an LF diet, but energy intake and body weight for *Pomc*^tm1/tm1^ mice compared with *Pomc*^wt/wt^ mice fed an HF diet significantly increased by 10.4% (10.74 ± 0.27 vs 9.73 ± 0.13 kcal/mouse/d; NS) and 16.1% (20.60 ± 0.45 vs 17.75 ± 0.19 g; *P* < 0.001), respectively (Table 2). For older female mice (14 weeks postweaning), energy intake and body weight for *Pomc*^tm1/tm1^ mice increased compared with *Pomc*^wt/wt^ mice by 2.5% (74.23 ± 0.57 vs 72.43 ± 0.79 kcal/mouse/wk; NS) and 11.8% (23.76 ± 0.71 vs 21.26 ± 0.35 g; NS), respectively, when mice were fed an LF diet and by 2.7% (84.57 ± 2.36 vs 82.32 ± 2.36 kcal/mouse/wk; NS) and 31.7% (29.17 ± 1.04 vs 22.15 ± 0.19 g; *P* < 0.001), respectively, when mice were fed an HF diet (Table 2).

### Increased energy intake exceeds increased body weight gained for HF diet–induced young male *Pomc*^wt/wt^ mouse obesity and for HF diet–exacerbated young male and female *Pomc*^tm1/tm1^ mouse obesity

For male *Pomc*^w*t*/wt^ mice 28 days postweaning, energy intake and body weight significantly increased for HF compared with LF diet–fed mice by 20.3% (12.52 ± 0.50 vs 10.41 ± 0.08 kcal/mouse/d; *P* < 0.01) and 14.1% (25.58 ± 0.65 vs 22.42 ± 0.31 g; *P* < 0.01), respectively, whereas energy intake and body weight for male *Pomc*^tm1/tm1^ mice fed an HF diet compared with an LF diet significantly increased by 16.9% (13.62 ± 0.33 vs 11.65 ± 0.30 kcal/mouse/d; *P* < 0.01) and 11.2% (27.66 ± 0.22 vs 24.88 ± 0.16 g; *P* < 0.01), respectively (Table 1).

For female *Pomc*^tm1/tm1^ mice at 28 days postweaning, energy intake and body weight significantly increased for HF compared with LF diet–fed mice by 17.0% (10.74 ± 0.27 vs 9.18 kcal/mouse/d; *P* < 0.0001) and 9.5% (20.60 ± 0.45 vs 18.81 ± 0.24 g; *P* < 0.0001), respectively. Energy intake and body weight were not different for female *Pomc*^wt/wt^ mice fed an LF or HF diet at 28 days postweaning (Table 2).

### Increased body weight gained exceeds increased energy intake for HF diet–induced older male *Pomc*^wt/wt^ mouse obesity and for HF diet–exacerbated older male and female *Pomc*^tm1/tm1^ mouse obesity

For older male *Pomc*^w*t*/wt^ mice at 14 weeks postweaning, energy intake and body weight for HF compared with LF diet–fed mice significantly increased by 17.7% (96.56 ± 3.26 vs 82.07 ± 2.48 kcal/mouse/wk; *P* < 0.01) and 22.8% (36.63 ± 1.15 vs 29.82 ± 0.19 g; *P* < 0.01), respectively, whereas energy intake and body weight for male *Pomc*^tm1/tm1^ mice fed an HF diet compared with an LF diet significantly increased 19.9% (104.26 ± 1.46 vs 86.97 ± 1.21 kcal/mouse/wk; *P* < 0.01) and 31.5% (43.62 ± 0.42 vs 33.17 ± 0.30 g; *P* < 0.01), respectively (Table 1).

For older female *Pomc*^w*t*/wt^ mice at 14 weeks postweaning, energy intake for HF compared with LF diet–fed mice significantly increased by 13.7% (82.32 ± 2.36 vs 72.43 ± 0.79 kcal/mouse/wk; *P* < 0.01) whereas body weight (22.15 ± 0.19 vs 21.26 ± 0.35 g) did not increase (Table 2). For older female *Pomc*^tm1/tm1^ mice at 14 weeks postweaning, energy intake for HF compared with LF diet–fed mice significantly increased by 13.9% (84.57 ± 2.36 vs 74.23 ± 0.57 kcal/mouse/d; *P* < 0.0001) whereas body weight significantly increased 22.8% (29.17 ± 1.04 vs 23.76 ± 0.71 g; *P* < 0.0001) (Table 2).

### Male and female *Pomc*^tm1/tm1^ mice compared with *Pomc*^wt/wt^ mice have significantly larger abdominal organs when mice are fed an HF diet

*Pomc* ^tm1/tm1^ mice have significantly increased abdominal organ weights compared with *Pomc*^wt/wt^ mice, but only when they are chronically fed HF diet. Male *Pomc*^tm1/tm1^ compared with *Pomc*^wt/wt^ mice had significantly increased heart (*P* < 0.05), liver (*P* < 0.01), pancreas (*P* < 0.001), and spleen (*P* < 0.05) weights (32). Similarly, female *Pomc*^tm1/tm1^ mice compared with *Pomc*^wt/wt^ mice exhibited significantly increased liver (*P* < 0.001) and spleen (*P* < 0.05) weights (32).

### An HF diet induces increased male *Pomc*^tm1/tm1^ mouse heart, liver, and pancreas weights and female *Pomc*^tm1/tm1^ mouse pancreas weights

Chronic HF diet–induced increases in male and female *Pomc*^tm1/tm1^ mouse body weight compared with *Pomc*^wt/wt^ mice are not only a result of increased fat mass but also increased mass for several abdominal organ weights. A chronic HF diet compared with an LF diet significantly increased male *Pomc*^tm1/tm1^ heart (*P* < 0.05), liver (*P* < 0.05), and pancreas (*P* < 0.001) weights (32). Similarly, a chronic HF diet compared with an LF diet significantly increased female *Pomc*^tm1/tm1^ pancreas weight (*P* < 0.05). In contrast, an HF diet did not significantly increase the weights for these organs in male or female *Pomc*^wt/wt^ mice (32). Brain, stomach, and kidney weights were not affected by an HF diet for male or female *Pomc*^wt/wt^ and female *Pomc*^tm1/tm1^ mice compared with an LF diet.

### A chronic HF diet exacerbates hyperglycemia in male but not female *Pomc*^tm1/tm1^ mice, without altering fasting plasma insulin or adiponectin, and a chronic HF diet reduces plasma adiponectin only in female *Pomc*^wt/wt^ mice

**Figure 4.**
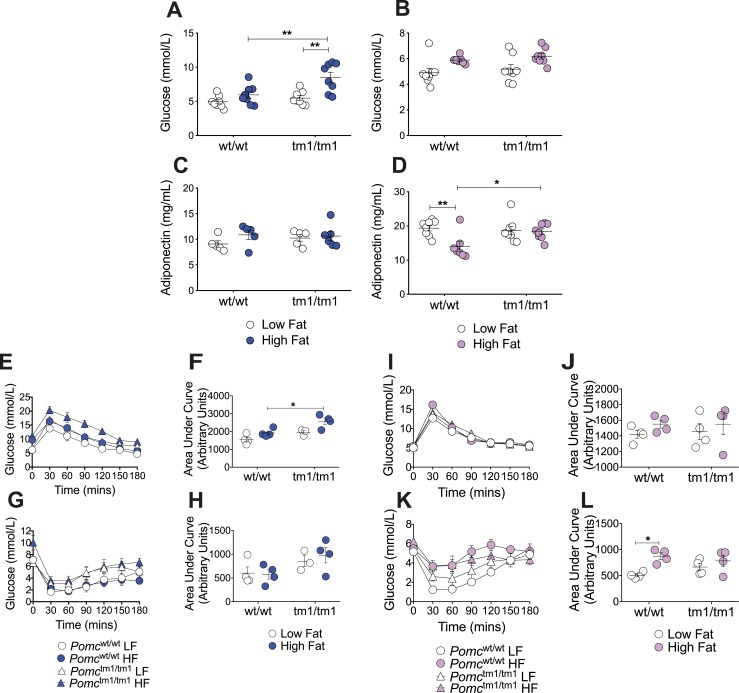
A chronic HF diet exacerbates hyperglycemia in male, but not female, *Pomc*^tm1/tm1^ mice and reduces plasma adiponectin while inducing insulin resistance in female *Pomc*^wt/wt^ mice, but not in male or female *Pomc*^tm1/tm1^ mice. (A–D) Fasting (A and B) blood glucose and (C and D) plasma adiponectin measured at 21 to 23 wk postweaning for (A and C) male and (B and D) female mice fed either an LF or HF diet from weaning. Data are shown as mean ± SEM (n = 5 to 8). Significant differences were determined using a two-way ANOVA and Tukey *post hoc* test. *P < 0.05; **P < 0.01. (E, F, I, and J) GTTs and (G, H, K, and L) ITTs were performed on (E, F, G, and H) male and (I, J, K, and L) female mice aged 17 to 18 wk. GTTs and ITTs were performed on the same mice with 1 wk between tests. Data are shown as mean ± SEM (n = 3 to 4). Significant differences for area under the curve were determined using a one-way ANOVA and Tukey *post hoc* test. *P < 0.05.

An HF diet compared with an LF diet significantly increased fasting blood glucose in male *Pomc*^tm1/tm1^ mice but not in male *Pomc*^wt/wt^ mice (*P* < 0.01) (Fig. 4A). Fasting blood glucose was significantly increased for male *Pomc*^tm1/tm1^ male mice compared with male *Pomc*^wt/wt^ mice, but only when they were fed an HF diet (*P* < 0.01) (Fig. 4A). Female *Pomc*^tm1/tm1^ mice did not exhibit increased fasting blood glucose levels compared with female *Pomc*^wt/wt^ mice when fed either an LF or HF diet (Fig. 4B).

Fasting plasma insulin was not significantly affected by an HF diet compared with an LF diet for either male *Pomc*^wt/wt^ or male *Pomc*^tm1/tm1^ mice (32). Furthermore, fasting plasma insulin was not significantly different between male *Pomc*^wt/wt^ and *Pomc*^tm1/tm1^ mice fed either an LF or HF diet (32). Fasting plasma insulin measured for female *Pomc*^wt/wt^ and *Pomc*^tm1/tm1^ mice on an LF or HF diet was undetectable in both ELISA and Milliplex MAP kit.

Fasting plasma adiponectin was not significantly affected by an HF compared with an LF diet for either male *Pomc*^wt/wt^ or male *Pomc*^tm1/tm1^ mice (Fig. 4C). In contrast with males, an HF diet compared with an LF diet significantly reduced fasting plasma adiponectin in female *Pomc*^wt/wt^ mice (*P* < 0.01) without having any effect on female *Pomc*^tm1/tm1^ mice (Fig. 4D). This likely explains why female *Pomc*^tm1/tm1^ mice fed an HF diet had significantly increased adiponectin levels compared with female *Pomc*^wt/wt^ mice fed an HF diet (*P* < 0.05) (Fig. 4D).

### A chronic HF diet induces glucose intolerance in male *Pomc*^tm1/tm1^ mice, but not in male *Pomc*^wt/wt^, female *Pomc*^wt/wt^, or female *Pomc*^tm1/tm1^ mice, and a chronic HF diet induces insulin resistance in female *Pomc*^wt/wt^ mice, but not in male *Pomc*^wt/wt^, male *Pomc*^tm1/tm1^, or female *Pomc*^tm1/tm1^ mice

A chronic HF diet induced impaired GTTs (*P* < 0.05) in male *Pomc*^tm1/tm1^ mice compared with male *Pomc*^wt/wt^ mice (Fig. 4E and 4F) with no significant effect on ITTs (Fig. 4G and 4H). In contrast to males, GTTs and ITTs in female *Pomc*^tm1/tm1^ mice were not significantly different from GTTs or ITTs in female *Pomc*^wt/wt^ mice when mice were fed either an LF or HF diet (Fig. 4I, 4J, 4K, and 4L). Surprisingly, although female *Pomc*^wt/wt^ mice are resistant to weight gain when fed an HF diet, there were significantly impaired ITTs in female *Pomc*^wt/wt^ mice but not in female *Pomc*^tm1/tm1^ mice fed an HF diet, compared with female *Pomc*^wt/wt^ mice fed an LF diet (*P* < 0.05) (Fig. 4K and 4L).

### Desacetyl-*α*-MSH or *α*-MSH administered centrally reduces male *Pomc*^tm1/tm1^ mouse body weight and fat mass when mice are fed an HF or LF diet

**Figure 5.**
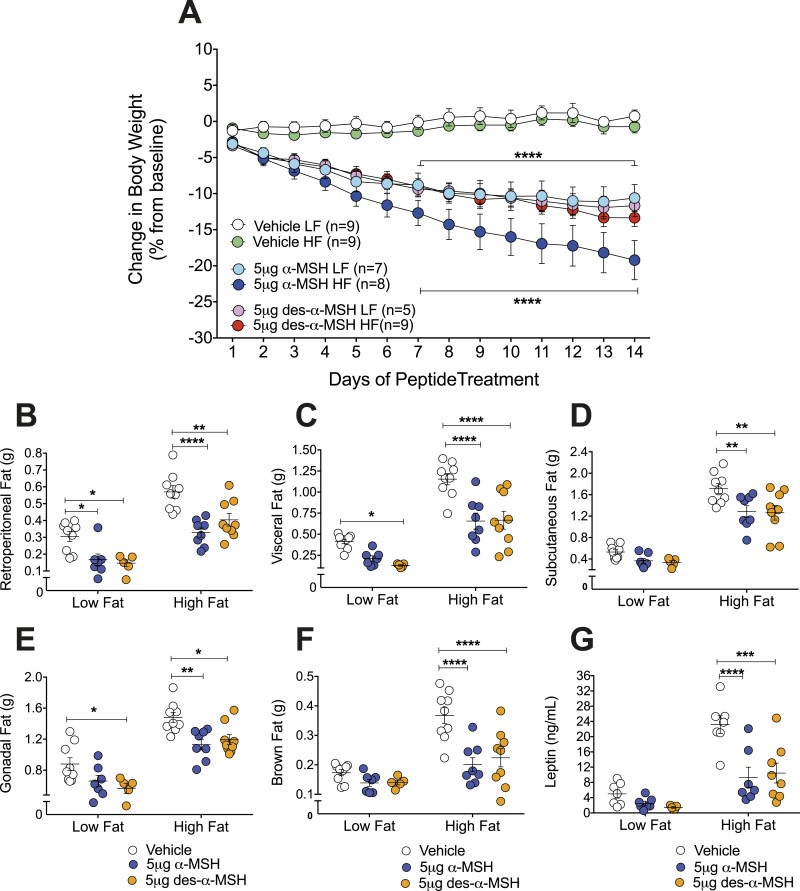
Central *α*-MSH or desacetyl-*α*-MSH treatment reduces male *Pomc*^tm1/tm1^ mouse body weight and fat mass when mice are fed either an LF or HF diet. (A) ICV administration of *α*-MSH or desacetyl-*α*-MSH compared with vehicle treatment reduces male *Pomc*^tm1/tm1^ mouse body weight when mice are fed either an LF or HF diet. The ages and body weights of the mice at the start of treatment are shown in an online repository (32). Vehicle or peptide dose (5 μg/25 g mouse body weight on day 1/d) was continuously administered during 14 d. Combined data are shown as mean ± SEM for two independent experiments. Significant differences were determined using a two-way repeated measures ANOVA and Dunnett *post hoc* analysis. Vehicle LF vs *α*-MSH LF, vehicle LF vs desacetyl-*α*-MSH LF, vehicle HF vs *α*-MSH HF, and vehicle HF vs desacetyl-*α*-MSH HF are shown. ****P < 0.0001. Significant differences not shown are day 2, vehicle LF vs desacetyl-*α*-MSH LF (*P* < 0.05); day 3, vehicle LF vs *α*-MSH LF (*P* < 0.01) or desacetyl-*α*-MSH LF (*P* < 0.05) and vehicle HF vs *α*-MSH HF (*P* < 0.05); day 4, vehicle LF vs *α*-MSH LF (*P* < 0.001) or desacetyl-*α*-MSH LF (*P* < 0.01) and vehicle HF vs *α*-MSH HF (*P* < 0.001) or desacetyl-*α*-MSH HF (*P* < 0.05); day 5, vehicle LF vs *α*-MSH LF (*P* < 0.0001) or desacetyl-*α*-MSH (*P* < 0.001) and vehicle HF vs *α*-MSH HF (*P* < 0.0001) or desacetyl-*α*-MSH HF (*P* < 0.01); and day 6, vehicle LF vs *α*-MSH LF (*P* < 0.0001) or desacetyl-*α*-MSH (*P* < 0.001) and vehicle HF vs *α*-MSH HF (*P* < 0.0001) or desacetyl-*α*-MSH HF (*P* < 0.01). (B–F) Fat pad weights for male *Pomc*^tm1/tm1^ mice fed either an LF or HF diet after 14 d of ICV administration of vehicle, *α*-MSH, or desacetyl-*α*-MSH. Data are shown as mean ± SEM (n = 5 to 9). Significant differences are shown between vehicle and peptide treatment as determined using a two-way ANOVA and Tukey *post hoc* analysis. *P < 0.05; **P < 0.01; ***P < 0.001; ****P < 0.0001. (G) Nonfasting plasma leptin of male *Pomc*^tm1/tm1^ mice fed an LF or HF diet after 14 d of ICV administration of vehicle, *α*-MSH, or desacetyl-*α*-MSH. Data are shown as mean ± SEM (n = 5 to 8). Significant differences between vehicle and peptide treatment were determined using a two-way ANOVA and Tukey *post hoc* analysis. ***P < 0.01; ****P < 0.0001.

Body weight of male *Pomc*^tm1/tm1^ mice fed an LF diet from weaning was significantly reduced by ∼11% to 12% following 14 days of continuous intracerebroventricular (ICV) administration of either 5 μg of desacetyl-*α*-MSH/25 g of mouse body weight/d or 5 μg of *α*-MSH/25 g of mouse body weight/d, compared with vehicle-treated age-matched control *Pomc*^tm1/tm1^ mice (*P* < 0.0001) (Fig. 5A). Body weight of male *Pomc*^tm1/tm1^ mice fed an HF diet from weaning was significantly reduced by ∼13% and ∼19% following 14 days of continuous ICV administration of either 5 μg of desacetyl-*α*-MSH/25 g of mouse body weight/d or 5 μg of *α*-MSH/25 g of mouse body weight/d, respectively, compared with vehicle-treated, age-matched control *Pomc*^tm1/tm1^ mice (*P* < 0.0001) (Fig. 5A).

The reductions in male *Pomc*^tm1/tm1^ mouse body weight are mirrored by reductions in adipose tissue weights and plasma leptin levels. Centrally administered *α*-MSH or desacetyl-*α*-MSH significantly reduced retroperitoneal, visceral, subcutaneous, gonadal, and brown fat masses for male *Pomc*^tm1/tm1^ mice fed an HF diet (Fig. 5B, 5C, 5D, 5E, and 5F). Centrally administered *α*-MSH significantly reduced retroperitoneal fat mass (Fig. 5B) and desacetyl-*α*-MSH significantly reduced retroperitoneal, visceral, and gonadal fat masses for male *Pomc*^tm1/tm1^ mice fed an LF diet (Fig. 5B, 5C, and 5F). Centrally administered desacetyl-*α*-MSH or *α*-MSH also significantly reduced nonfasting plasma leptin (*P* < 0.001) for male *Pomc*^tm1/tm1^ mice fed an HF diet (Fig. 5G).

## Discussion

Our discovery that chronic HF diet exacerbates male and female *Pomc*^tm1/tm1^ mouse obesity demonstrates that the obese state caused by the absence of *α*-MSH and desacetyl-*α*-MSH leaves mice vulnerable to an obesogenic environment. Our data highlight distinct mechanisms underlying HF diet–induced and impaired melanocortin signaling-induced mouse obesity. We also show, to our knowledge for the first time, that desacetyl-*α*-MSH and *α*-MSH are required for sexually dimorphic chronic HF diet–induced obesity in C57BL/6J mice. Female *Pomc*^wt/wt^ mice but not *Pomc*^tm1/tm1^ mice are protected from HF diet–induced obesity. Neither male *Pomc*^wt/wt^ mice nor *Pomc*^tm1/tm1^ mice are protected from HF diet–induced obesity. Furthermore, we show that either desacetyl-*α*-MSH *ο*r *α*-MSH continuously administered during 14 days into the brains of male *Pomc*^tm1/tm1^ mice reverses chronic HF diet–exacerbated obesity.

The data presented in the present study on *Pomc*^tm1/tm1^ mice fed an LF diet are consistent with our previous results for *Pomc*^tm1/tm1^ mice fed regular chow diet, demonstrating that male and female *Pomc*^tm1/tm1^ mice develop obesity associated with increased body length (8). However, previously we showed that male and female *Pomc*^tm1/tm1^ mice are hyperphagic compared with *Pomc*^wt/wt^ mice, and in the present study we show that only male *Pomc*^tm1/tm1^ mice are hyperphagic compared with *Pomc*^wt/wt^ mice when fed an LF or HF diet. A number of factors likely contribute to the difference in food intake between the two studies for female mice. The previous study measured food intake for <1 week whereas the present study monitored food intake during 16 weeks. The environmental conditions between the two studies varied, including different animal facilities, regular chow vs an LF diet, metabolic vs standard cages, and individual vs group housing, all of which can affect mouse food intake. The known underlying C57BL/6J mouse sexually dimorphic metabolic differences (14, 27, 36) likely explain why we only observed hyperphagic differences for females between the two studies.

In contrast to an acute HF diet (8), a chronic HF diet compared with an LF diet does not induce hyperphagia in male or female *Pomc*^tm1/tm1^ mice. HF diet exacerbation of male *Pomc*^tm1/tm1^ mouse obesity is a result of increased energy intake because male *Pomc*^tm1/tm1^ mice are hyperphagic on either an LF or HF diet compared with male *Pomc*^wt/wt^ mice, and their energy intake fully accounts for their increased body weight gained during 14 to 28 days postweaning. However, HF diet exacerbation of female *Pomc*^tm1/tm1^ mouse obesity is not a result of increased energy intake, nor is it a result of increased feed efficiency. Our data point to reduced energy expenditure being the major contributor to young female *Pomc*^tm1/tm1^ mouse obesity compared with *Pomc*^wt/wt^ mice when mice are fed an HF diet. Furthermore, the greater increase in body weight gained relative to the increase in energy intake for older female *Pomc*^tm1/tm1^ mice compared with *Pomc*^wt/wt^ mice similarly points to older female *Pomc*^tm1/tm1^ mice having reduced energy expenditure compared with *Pomc*^wt/wt^ mice, regardless of whether they are fed an LF or HF diet. Therefore, hyperphagia and energy expenditure appear to be sexually dimorphic phenotypes for *Pomc*^tm1/tm1^ mice. Hyperphagia is a major contributor to male *Pomc*^tm1/tm1^ mouse obesity although reduced energy expenditure appears to be a major contributor to female *Pomc*^tm1/tm1^ obesity.

Male *Pomc*^tm1/tm1^ mouse obesity is exacerbated by a chronic HF diet because mice eat the same quantity of an HF diet as an LF diet, but they obtain higher energy intake eating the HF diet. Although female *Pomc*^tm1/tm1^ mice retain some ability to sense a high-caloric diet and respond by decreasing food intake as observed for female *Pomc*^wt/wt^ mice, female *Pomc*^tm1/tm1^ mouse obesity is exacerbated when they are fed an HF diet and their body weight gained is greater than their energy intake. Feed efficiency is not increased, and therefore reduced energy expenditure must be a major contributor to chronic HF diet–exacerbated female *Pomc*^tm1/tm1^ obesity.

Fat depot weights for *Pomc*^tm1/tm1^ compared with *Pomc*^wt/wt^ mice are also sexually dimorphic when mice are fed an HF diet. Male, but not female, *Pomc*^tm1/tm1^ mice compared with *Pomc*^wt/wt^ mice chronically fed an HF diet exhibit preferential fat storage in visceral, subcutaneous, and brown adipose depots over retroperitoneal and gonadal adipose depots. In contrast, female *Pomc*^tm1/tm1^ mice chronically fed an HF diet do not show any fat depot preference because significant increases are observed for all five fat depots studied, compared with female *Pomc*^wt/wt^ mice. Distinct effects for *Pomc*^tm1/tm1^ obesity and chronic HF diet–induced obesity on fat cell size are also observed. A chronic HF diet compared with an LF diet significantly increases male *Pomc*^wt/wt^ mouse retroperitoneal fat cell size, whereas male *Pomc*^tm1/tm1^ mice compared with *Pomc*^wt/wt^ mice fed an HF diet exhibit increased subcutaneous fat cell size. In contrast to sexually dimorphic effects for fat depot weights, male and female *Pomc*^tm1/tm1^ mice both exhibit significantly increased abdominal organ weights when mice are fed an HF diet.

Differential effects on fat depots are evidence that a chronic HF diet could alter glucose homeostasis in *Pomc*^tm1/tm1^ mice, and altered glucose homeostasis could be a sexually dimorphic phenotype for *Pomc*^tm1/tm1^ mice. Indeed, in the present study we show that a chronic HF diet exacerbates hyperglycemia and impairs GTTs in male, but not female, *Pomc*^*t*m1/tm1^ mice. Subcutaneous fat, compared with retroperitoneal fat, is considered “good fat” (33), and therefore male *Pomc*^tm1/tm1^ mouse hyperglycemia and impaired GTTs would not be expected to predict risk of type 2 diabetes for male *Pomc*^tm1/tm1^ mice chronically fed an HF diet. As predicted, we did not observe type 2 diabetes because the ITT was not impaired for either male or female *Pomc*^tm1/tm1^ mice compared with Pomc^wt/wt^ mice fed an LF or HF diet.

Surprisingly, we show in the present study that sexually dimorphic diet-induced obesity in C57BL/6J mice is associated with insulin resistance in female mice. Not only are female *Pomc*^wt/wt^ mice resistant to HF diet–induced obesity, but they exhibit significantly impaired ITTs and significantly reduced plasma adiponectin levels when fed an HF diet compared with an LF diet.

Overall our data add to the growing body of knowledge about sexually dimorphic effects associated with melanocortin obesity (10, 37–41) and with HF diet–induced obesity in C57BL/6J mice. Although mechanisms explaining sexually dimorphic melanocortin obesity and associated altered metabolic effects are not known, impaired adaptive thermogenesis has been identified as a mechanism explaining sexually dimorphic chronic HF diet–induced C57BL/6J mouse obesity, when mice are fed an HF diet from weaning (27). Female mice are resistant to developing HF diet–induced obesity when housed at 20 to 23°C but not when housed at thermoneutrality (30 to 32°C), whereas male mice develop HF diet–induced obesity at either housing temperature (27). Giles *et al.* (27) have shown that adaptive thermogenesis is triggered in female mice when they sense a high-caloric diet and they burn the extra calories rather than store excess calories as fat. Alternatively, male mice are either not able to sense a high-caloric diet or they are not able to trigger adaptive thermogenesis, or both; they store excess calories as fat and become obese. This identifies sensing a high-caloric diet and responding by turning on adaptive thermogenesis to maintain body weight as sexually dimorphic responses, at least in C57BL/6J mice. In our study, we show that *α*-MSH and desacetyl-*α*-MSH are required for sexually dimorphic HF diet–induced C57BL/6J mouse obesity and, by association, *α*-MSH and desacetyl-*α*-MSH are required for female mice sensing a high-caloric diet and triggering adaptive thermogenesis. However, an additional mechanism is needed to explain male *Pomc*^tm1/tm1^ mouse hyperphagia compared with *Pomc*^wt/wt^ mice fed either an LF or HF diet.

Collectively, our data suggest that desacetyl-*α*-MSH and *α*-MSH play salutary roles in the protection against HF diet–induced mouse obesity in a sex-dependent manner. The molecular mechanisms that mediate sexually dimorphic melanocortin obesity and altered glucose homeostasis are unknown, but 17*β*-estradiol (E2) likely plays a role. Current evidence suggests that E2 interacts with the melanocortin system to regulate energy homeostasis, but it is not clear whether it leads to the sexually dimorphic phenotype observed in this study for *Pom*c^tm1/tm1^ mice. Both male and female estrogen receptor *α* global knockout mice develop obesity (42). E2 activates POMC neurons, and E2 replacement in rodents prevents ovariectomized-induced obesity by decreasing food intake and increasing energy expenditure (43). However, whereas estrogen receptor *α* in arcuate nucleus POMC neurons can regulate feeding, E2 appears to regulate energy expenditure through non–POMC-expressing arcuate nucleus neurons (43, 44). Burke *et al.* (38) discovered a sex difference for a subset of arcuate nucleus POMC neurons expressing 5-hydroxytryptamine 2c receptor (5-HT_2c_R). Restoration of POMC expression only in arcuate nucleus POMC neurons expressing 5-HT_2c_R rescued hyperphagia in both male and female arcuate nucleus POMC null mice, but it only rescued energy expenditure in male but not female arcuate nucleus POMC-null mice (38). Therefore, the mechanisms for sexually dimorphic HF diet–induced and impaired melanocortin-induced reductions in energy expenditure may involve arcuate nucleus POMC neurons expressing 5-HT_2c_R, a subset of 5-HT_2c_R-expressing POMC neurons, or a POMC neuron subtype yet to be functionally characterized (45).

Our data support that a chronic HF diet compared with an LF diet increases energy expenditure for young male *Pomc*^wt/wt^ and *Pomc*^tm1/tm1^ mice, but it decreases energy expenditure for older male *Pomc*^wt/wt^ and *Pomc*^tm1/tm1^ mice. Similarly, our data support that a chronic HF diet compared with an LF diet increases energy expenditure for young female *Pomc*^tm1/tm1^ mice, but it decreases energy expenditure for older female *Pomc*^tm1/tm1^ mice. The apparent decreased energy expenditure associated with older mice feeding on an HF diet could be a consequence of the obese phenotype. Female *Pomc*^wt/wt^ are resistant to HF diet–induced obesity, and our data support that a chronic HF diet compared with an LF diet increases energy expenditure for both young and older female *Pomc*^wt/wt^ mice.

In summary, male *Pomc*^tm1/tm1^ mice develop obesity because they are hyperphagic, and their obesity is exacerbated when they are fed an HF diet because they do not sense and respond appropriately to increased caloric intake, to defend their body weight. Female *Pomc*^tm1/tm1^ mice develop obesity because they appear to have reduced energy expenditure, and their obesity is exacerbated when they are fed an HF diet because they have impaired sensing of a high-caloric diet and apparent impaired adaptive thermogenesis. Importantly, we show in the present study that continuous infusion of either desacetyl-*α*-MSH or *α*-MSH centrally to male *Pomc*^tm1/tm1^ mice chronically fed an HF diet reverses obesity. Desacetyl-*α*-MSH is much more abundant than *α*-MSH in the rodent brain (46, 47) and in the human brain (48); therefore, both desacetyl-*α*-MSH and *α*-MSH likely play important roles in regulating body weight and fat mass in the presence of an obesogenic diet.

## Conclusion

We show in the present study that understanding the impact of a chronic HF diet on the development of impaired melanocortin signaling driven obesity unravels mechanisms controlling energy homeostasis. Of note, we show sexually dimorphic physiological roles for desacetyl-*α*-MSH and *α*-MSH regulating sensitivity to caloric intake by regulating food intake and energy expenditure. This suggests that understanding how endogenous desacetyl-*α*-MSH and *α*-MSH are regulated and signal *in vivo*, in the presence of obesogenic diets, could lead to future prevention or treatment of obesity.

## Abbreviations:

5-HT_2c_R: 5-hydroxytryptamine 2c receptor
CV: coefficient of variation
E2: 17*β*-estradiol
GTT: glucose tolerance test
HF: high-fat
ICV: intracerebroventricular(ly)
ITT: insulin tolerance test
LF: low-fat
Mc3r: melanocortin-3 receptor
Mc4r: melanocortin-4 receptor
NS: not significant
POMC: pro-opiomelanocortin
WT: wild-type
